# Peripheral T-lymphocytes express WNT7A and its restoration in leukemia-derived lymphoblasts inhibits cell proliferation

**DOI:** 10.1186/1471-2407-12-60

**Published:** 2012-02-07

**Authors:** Alejandra B Ochoa-Hernández, Moisés Ramos-Solano, Ivan D Meza-Canales, Beatriz García-Castro, Mónica A Rosales-Reynoso, Judith A Rosales-Aviña, Esperanza Barrera-Chairez, Pablo C Ortíz-Lazareno, Georgina Hernández-Flores, Alejandro Bravo-Cuellar, Luis F Jave-Suarez, Patricio Barros-Núñez, Adriana Aguilar-Lemarroy

**Affiliations:** 1 División de Genética, Centro de Investigación Biomédica de Occidente (CIBO), Instituto Mexicano del Seguro Social (IMSS), Guadalajara, Jalisco, Mexico; 2 Programa de Doctorado en Genética Humana, Universidad de Guadalajara, Guadalajara, Jalisco, Mexico; 3 División de Inmunología, CIBO-IMSS, Guadalajara, Jalisco, Mexico; 4 División de Medicina Molecular, CIBO-IMSS, Guadalajara, Jalisco, Mexico; 5 Servicio de Hematología, Hospital Civil de Guadalajara Fray Antonio Alcalde, Universidad de Guadalajara, Guadalajara, Jalisco, Mexico; 6 División de Inmunología, Centro de Investigación Biomédica de Occidente, Instituto Mexicano del Seguro Social, Sierra Mojada No. 800, Col. Independencia, 44340 Guadalajara, Jalisco, Mexico

**Keywords:** WNT7A, Wnt signaling, Leukemia, Anti-proliferative, Non-canonical pathway

## Abstract

**Background:**

WNT7a, a member of the Wnt ligand family implicated in several developmental processes, has also been reported to be dysregulated in some types of tumors; however, its function and implication in oncogenesis is poorly understood. Moreover, the expression of this gene and the role that it plays in the biology of blood cells remains unclear. In addition to determining the expression of the *WNT7A *gene in blood cells, in leukemia-derived cell lines, and in samples of patients with leukemia, the aim of this study was to seek the effect of this gene in proliferation.

**Methods:**

We analyzed peripheral blood mononuclear cells, sorted CD3 and CD19 cells, four leukemia-derived cell lines, and blood samples from 14 patients with Acute lymphoblastic leukemia (ALL), and 19 clinically healthy subjects. Reverse transcription followed by quantitative Real-time Polymerase chain reaction (qRT-PCR) analysis were performed to determine relative *WNT7A *expression. Restoration of WNT7a was done employing a lentiviral system and by using a recombinant human protein. Cell proliferation was measured by addition of WST-1 to cell cultures.

**Results:**

WNT7a is mainly produced by CD3 T-lymphocytes, its expression decreases upon activation, and it is severely reduced in leukemia-derived cell lines, as well as in the blood samples of patients with ALL when compared with healthy controls (*p *≤0.001). By restoring *WNT7A *expression in leukemia-derived cells, we were able to demonstrate that WNT7a inhibits cell growth. A similar effect was observed when a recombinant human WNT7a protein was used. Interestingly, restoration of *WNT7A *expression in Jurkat cells did not activate the canonical Wnt/β-catenin pathway.

**Conclusions:**

To our knowledge, this is the first report evidencing quantitatively decreased *WNT7A *levels in leukemia-derived cells and that *WNT7A *restoration in T-lymphocytes inhibits cell proliferation. In addition, our results also support the possible function of *WNT7A *as a tumor suppressor gene as well as a therapeutic tool.

## Background

The Wnt signaling pathway describes a complex network of proteins involved in differentiation, proliferation, migration, and cell polarity, which play important roles during embryonic development, tissue regeneration, and in homeostatic mechanisms [1,2]. Wnt molecules are a highly conserved group of secreted cysteine-rich lipoglycoproteins that work as signaling molecules. Nineteen different Wnt family members have been described in humans to date. The binding of these ligands to its receptor complex (Frizzled/LRP-5/6) leads to activation of the pathway [1,3]. Distinct sets of Wnt and Frizzled ligand-receptor pairs can activate different pathways and lead to unique cellular response [3,4]. Wnt signals are transduced through at least three different intracellular pathways: Wnt/β-catenin, also known as canonical pathway; Wnt/Ca++, and the Planar cell polarity (Wnt/JNK) pathway [1,3,5]. In the canonical pathway, receptor activation leads to stabilization of β-catenin by inhibiting the phosphorylation activity of the Glycogen synthase kinase (GSK)-3β. Unphosphorylated β-catenin accumulates in the cytoplasm and then translocates into the nucleus, activating target gene expression through a complex network of co-receptors (TCF/LEF transcription factors) and repressors (Groucho) [6-8]. The Wnt/β-catenin pathway is involved in the self-renewal and proliferation of hematopoietic stem cells and has been also implicated in numerous types of cancers [7,9,10]. Dysregulation of this pathway is a hallmark of several types of tumors [7,11-13].

Leukemic cells are highly heterogeneous, and their mechanisms of tumorigenesis are poorly understood. Recently, dysregulation of the Wnt signaling pathway has been implicated in the pathogenesis of some leukemia types [14-17]. Moreover, different expression profiles of some *WNT *genes and their related signaling molecules have been reported in hematological cancers [12,18-22]. However, there are limited numbers of studies regarding the role of WNT7a, both in normal and in leukemia-derived cells [19,23,24].

Because our group has observed strongly decreased expression of *WNT7A *in different tumor-derived cell lines (unpublished data), we have focused our attention on the expression of *WNT7A *in normal peripheral blood cells, in leukemia-derived cell lines, and in patients with Acute lymphoblastic leukemia (ALL).

## Methods

### Ethics statement

Fourteen peripheral blood samples from patients with leukemia were collected from the Hospital Civil Fray Antonio Alcalde and blood samples from healthy volunteers at the Instituto Mexicano del Seguro Social (IMSS) Blood Bank after approval by the Ethical and Research Committee No. 1305 of the Centro de Investigación Biomédica de Occidente (CIBO) - IMSS (project approval numbers 1305-2006-07 and 1305-2010-2). Written informed consent from patients and healthy volunteers (following local Ethics Committee guidelines and international norms) was also required prior to blood sample collection.

### Cell line culture

The human leukemia-derived cell lines Jurkat, K562, CEM, HL60, and the BJAB cells (lymphoma-derived B cells) were used as study model. Jurkat and CEM possess a lymphoblastic phenotype, whereas K562 and HL60 have a myeloid origin. Cells were cultured in RPMI-1640 medium supplemented with 10% Fetal bovine serum (FBS), penicillin (100 U/mL), and streptomycin (100 μg/mL) at 37°C in a humidified atmosphere of 5% CO_2_. All products mentioned previously were obtained from the GIBCO™ Invitrogen Corporation.

### Sorting of CD3- and CD19-positive cells

Peripheral blood mononuclear cells (PBMC) obtained from five healthy volunteers (12 mL of peripheral blood) were isolated by density-gradient centrifugation with Ficoll-Paque™ PLUS (GE Healthcare). PBMC included lymphocytes, monocytes, macrophages, NK cells, and also basophils and dendritic cells. These cells can be extracted from whole blood using Ficoll, which separates the blood into a top layer of plasma, followed by a layer of PBMC and a bottom fraction of polymorphonuclear cells and erythrocytes. The PBMC were resuspended in PBS and stained with an anti-CD3 antibody (sc-1179-FITC, Santa Cruz Biotechnology) to select T-lymphocytes and with an anti-CD19 antibody (sc-19650-PE, Santa Cruz Biotechnology) to select B-lymphocytes. After incubation with both antibodies, cells were washed and positive cells for CD3 or CD19 were sorted on a FACSAria (Becton Dickinson).

### RNA isolation

Leukemia-derived cell lines were seeded at a density of 5 × 10^6 ^in 75-cm^3 ^flasks and harvested after 24 h for Total RNA extraction by using the PureLink™ Micro-to-Midi Total RNA Purification System (cat. no. 12183-018, Invitrogen) as described by the manufacturers. RNA from PBMC, CD3^+ ^and CD19^+^cells was also extracted from five healthy volunteers by this method and put together to create a representative sample group of each cell type for the qRT-PCR analysis.

Peripheral blood samples were collected from patients with ALL at the Hospital Civil Fray Antonio Alcalde. Additionally, 19 blood samples from healthy donors were collected as normal controls from the IMSS-Blood Bank (Table 1). Each sample (5 mL) was collected with EDTA as anticoagulant, mixed immediately after collection with 45 mL of RNA/DNA Stabilization Reagent for Blood/Bone marrow (Roche Applied Science), and stored at -80°C for conservation. From the stabilized samples, we took a volume corresponding to 6 × 10^5 ^leukocytes for each patient or control and utilized this for mRNA isolation via a two-step procedure through magnetic separation employing the mRNA Isolation Kit for blood/bone marrow (Roche Applied Science). mRNA was finally eluted from the magnetic pearls in 20 μL of water and stored at -80°C until use.

**Table 1:** Gender and Age of Control and Patients

Control ID	Gender	Age	Patient ID	Gender	Age	Diagnostic
**1**	M	33	**1**	M	38	ALL

**2**	M	26	**2**	M	82	ALL

**3**	F	54	**3**	M	56	ALL

**4**	F	34	**4**	F	46	ALL

**5**	F	68	**5**	F	32	ALL

**6**	M	51	**6**	F	36	ALL

**7**	F	43	**7**	F	56	ALL

**8**	F	24	**8**	M	84	ALL

**9**	F	56	**9**	M	61	ALL

**10**	M	40	**10**	M	58	ALL

**11**	F	53	**11**	F	30	ALL

**12**	F	35	**12**	M	52	ALL

**13**	F	26	**13**	F	43	ALL

**14**	M	39	**14**	M	18	ALL

**15**	M	73	**15**	F	47	CLL

**16**	M	45	**16**	F	31	CLL

**17**	F	39	**17**	F	37	CLL

**18**	M	40	**18**	F	58	CLL

**19**	M	26	**19**	F	89	CLL

			**20**	F	56	AML

			**21**	F	82	AML

			**22**	M	40	AML

			**23**	M	55	AML

			**24**	F	33	CML

			**25**	M	35	CML

			**26**	M	46	CML

### cDNA synthesis

For cell lines, cDNA synthesis was performed from 5 μg of total RNA. For patients or healthy volunteers, we used the maximum volume of mRNA permitted in the kit (8 μL). cDNA was obtained by using the SuperScript™ III First-Strand Synthesis System primed with oligo(dT) (cat. no. 18080051, Invitrogen). The protocol was carried out as recommended by the manufacturers.

### Primer design and qRT-PCR assays

Primers were designed using Oligo-Primer Analysis Software, version 6.51 (Molecular Biology Insights, Inc., USA) from sequences obtained from the GenBank Nucleotide database of the NCBI website. Primers were synthesized by Invitrogen Corporation, USA. The primers sequence, the location of each primer, the gene symbol, the sequence accession number, the amplicon length, and the annealing temperature used are summarized in Table 2. The extension time used for each set of primers was set by dividing the amplicon size by 25 sec, because the polymerase has the capability to insert 25 bp per second.

**Table 2:** Information of the oligonucleotides used for the qRT-PCR analysis

	Gene Name	Sequence Accession Number	Primer Sequence	Primer Location (Exon	Prod. Length	T°a
**WNT7A**	Wingless-type MMTV integration site family, member 7A	NM_004625	F CAAAGAGAAGCAAGGCCAGTA R GTAGCCCAGCTCCCGAAACTG	707-730 (3) 962-983 (4)	277	60

**MYC**	V-myc myelocytomatosis viral oncogene homolog (avian)	NM_002467.4	F CCAGCGCCTTCTCTCCGTC R GGGAGGCGCTGCGTAGTTGT	1208-1226 (2) 1490-1509 (3)	302	60

**JUN**	Jun proto-oncogene	NM_002228.3	F TGGAAAGTACTCCCCTAACCT R CTGAAACATCGCACTATCCTT	2786-2806 (1) 3015-3035 (1)	250	60

**FRA-1**	FOS-like antigen 1	NM_005438.3	F AGGAACCGGAGGAAGGAACTG R TGCCACTGGTACTGCCTGTGT	554-574 (3) 732-752 (4)	199	60

**AXIN2**	Axin 2	NM_004655.3	F AAAAAGGGAAATTATAGGTATTAC R CGATTCTTCCTTAGACTTTG	2678-2701 (10/11) 2935-2954 (11)	277	54

**GAPDH**	Glyceraldehyde-3-phosphate dehydrogenase	NM_002046.3	F CACTGCCACCCAGAAGACTGTG R TGTAGGCCATGAGGTCCACCAC	645-666 (8) 1072-1089 (9)	449	63

**RPL32**	Ribosomal protein L32	NM_000994.3	F GACTTGACAACAGGGTTCGTAG R ATTTAAACAGAAAACGTGCACA	213-234 (3) 511-532 (4)	320	60

**RPS18**	Ribosomal protein S18	NM_022551.2	F CGATGGGCGGCGGAAAA R CAGTCGCTCCAGGTCTTCACGG	105-121 (2) 366-387 (5)	283	58

**B2M**	Beta-2-microglobulin	NM_004048.2	F GAGGCTATCCAGCGTACTCCAA R CACACGGCAGGCATACTCAT	115-136 (1/2) 347-366 (2)	252	58

**ACTB**	Actin beta	NM_001101.3	F TCCGCAAAGACCTGTACG R AAGAAAGGGTGTAACGCAACTA	950-967 (5) 1226-1247 (6)	298	60

Gene expression analysis was achieved by qRT-PCR on the 1.5 LightCycler^® ^(Roche Diagnostics) using the LightCycler-FastStart DNA MasterPLUS SYBR Green I Kit (cat. no. 03515885001, Roche Applied Science) as recommended by the manufacturers. A standard curve with four serial dilution points and a negative control were included in each run. Relative expression was calculated with LightCycler software version 4.1 by taking *GAPDH*, *RPL32*, or *RPS18 *as reference genes.

Analysis in cell lines was performed by taking the values obtained from two independent RNA extractions in duplicate. In patients and healthy volunteers, experiments were performed three times in each individual sample.

In order to demonstrate that the reference genes selected and used in our cell model were appropriate, some samples were also tested with additional reference genes, as shown in Additional File 1.

### Δ CP analysis

For analysis of *WNT7A *expression in patients and in healthy volunteers, we used ΔCP to facilitate analysis by taking only intrinsic references from each sample. We compared the ΔCP values obtained for both groups, i.e., the *WNT7A *CP minus the reference gene CP from the same sample. *GAPDH *and *RPL32 *were used for normalization in this analysis. It is very important to point out that ΔCP is inversely proportional to the expression of the target gene.

### Isolation and culture of mononuclear cells

Peripheral blood mononuclear cells (PBMC) of healthy volunteers were isolated by density gradient using Ficoll-Paque PLUS (GE Healthcare, Sweden). Blood with anticoagulant was diluted 1:1 in PBS without MgCl_2 _and subsequently 1:1 in Ficoll-Paque PLUS. After 30-min centrifugation at 1,500 rpm, the cells' pellet was washed with PBS and resuspended in RPMI medium complemented with 10% FBS and antibiotics. Cells were led to growth at 37°C in an atmosphere of 5% CO_2 _in the presence or absence of phytohemagglutinin (PHA - 2 μg/mL).

### Treatment with WNT7a recombinant protein

Jurkat and PBMC were seeded at a density of 2 × 10^4 ^cells in a 96-well microtiter plate in 200 μL of RPMI medium. Recombinant human WNT7a (cat. no. 3008-WN/CF, R&D Systems) was resuspended first in sterile PBS and was afterward diluted 1:100 in the cell culture medium. Every 24 h the recombinant protein was added fresh to each well at a final concentration of 3 μg/mL; incubations at 37°C were performed for 24 and 48 h.

### Measurement of cell survival

After *WNT7A *overexpression or treatment with WNT7a recombinant protein, cell survival was determined by cleavage of tetrazolium salt WST-1 to formazan (cat. no. 11 644 807 001, Roche Applied Science) by reading the absorbance of treated and untreated cells at 440 nm on a microtiter plate reader (Synergy™ HT Multi-Mode Microplate Reader. Biotek. Winooski, VT, USA). The value of untreated cells was used as 100% cell survival.

### Cloning *WNT7A*

*WNT7A *Open reading frame (ORF) (GeneID: 7476; NM _004625) was amplified from human non-tumorigenic keratinocytes utilizing the Expand High Fidelity PCR System (cat. no. 11 732 650 001, Roche Applied Science) with the following set of primers: forward 5'-GGG ACT ATG AAC CGG AAA GC -3'; reverse: 5'- CGG GGC TCA CTT GCA CGT GTA C -3'. Afterward, the PCR product was cloned into the pCR2.1TOPO vector (Invitrogen). Construction was sequenced employing M13 Forward and Reverse primers (Invitrogen) with the BigDye^® ^Terminator Cycle Sequencing Kit (Applied Biosystems). After corroborating the *WNT7A *sequence with that reported in GenBank, *WNT7A *ORF was isolated from pCR2.1TOPO vector by EcoRI restriction and subcloned into the EcoRI site of the lentiviral expression vector pLVX-Puro or pLVX-Tight-Puro (Clontech Laboratories, USA).

### Lentivirus production and infection

To produce infectious viral particles, Lenti-X 293 T cells were transient-transfected by the Lentiphos HT/Lenti-X HT Packaging Systems with the lentiviral vectors pLVX-Puro or pLVX-WNT7A-Puro as described by the manufacturers (Clontech Laboratories. USA). Tet-Express Inducible Expression Systems were also used (pLVX-Tet-On Advanced and pLVX-Tight-WNT7A-Puro). After 48 h, supernatants were checked with Lenti-X GoStix (Clontech Laboratories. USA) to determine whether sufficient viral particles were produced before transducing target cells. Supernatants were filtered through a 0.45-μm PES filter to eliminate detached cells, were aliquoted, and subsequently stored at -80°C until use. Jurkat cells were transduced with 200 μL of supernatants obtained with pLVX-Puro or pLVX-WNT7A-Puro. RNA and protein extractions were obtained after 2 days of transduction and 1 week of puromycin selection (1 μg/mL). Cell proliferation was also measured by adding WST-1 to the culture cells at this point.

For inducible *WNT7A *expression in the leukemia-derived cell lines, cells were first transduced with the pLVX-Tet-On (regulator vector) and selected with G418 (cat. no. 631307, Clontech Laboratories, USA). Afterward, cells were transduced with the pLVX-Tight-WNT7A-Puro and selected with Puromycin for 2 weeks (1.5 μg/mL). After selection, cells were grown in the absence or presence of Doxycycline (Doxy) (750 ng/mL) to overexpress *WNT7A*.

### Western blot assays

Cells were harvested by scraping and were lysed with RIPA buffer by sonication (15 pulses, 50% amp). Extracts were incubated for 30 min at 4°C and obtained by centrifugation (14,000 rpm for 5 min at 4°C). Protein concentrations were determined using the Bio-Rad DC Protein Kit (cat. no. 500-0114 Protein DC - BioRad, Hercules, CA, USA) and 50 μg of whole-cell extracts were electrophoresed in 12% SDS-PAGE. Proteins were then transferred onto a PVDF membrane (Millipore) and incubated with 1% Western blocking reagent (cat no. 11921681001, Roche, Germany) to block nonspecific binding. Primary antibodies were incubated over night at 4°C and the secondary antibodies were incubated with the membrane for 2 h at RT, followed by chemiluminescent detection using Immobilon Western substrate (Millipore Corporation, USA) with the ChemiDoc XRS (Biorad Laboratories, USA). The primary antibodies used were: anti-β-Catenin (cat. no. SC-7199, Santa Cruz Biotechnology, USA), anti-WNT7A (cat. no. AF3008 and cat. no. K-15, from R&D and Santa Cruz Biotechnology, respectively), or anti-β-Actin (cat. no. SC-26361, Santa Cruz, Biothechnology).

### Apoptosis detection

Cell death was measured by flow cytometry using propidium iodide (cat. no. P4864, Sigma-Aldrich) and Annexin-V-FLUOS (cat. no. 1828681, Roche Applied Science) as recommended by these manufacturers. Cells were seeded at a density of 2.5 × 10^5 ^cells per flask in 10 mL RPMI medium with or without Doxycycline (750 ng/mL). After a 72 h incubation, cells were washed with PBS and incubated with Annexin and propidium iodide for 15 min; 10,000 events from each sample were analyzed in a FACS Aria cytometer (BD Biosciences).

### Statistical methods

Statistical analysis was performed with SPSS Statistics software version 17.0. Post-hoc tests (Tukey HSD, Bonferroni, and Dunnett 3 T) were utilized for multiple comparisons between groups, and one-way ANOVA was employed to compare the means among more than two different groups. Only *p *values < 0.05 were considered as significant.

## Results

### T-lymphocytes are the main cell population in peripheral blood that expresses *WNT7A*

**Figure 1:**
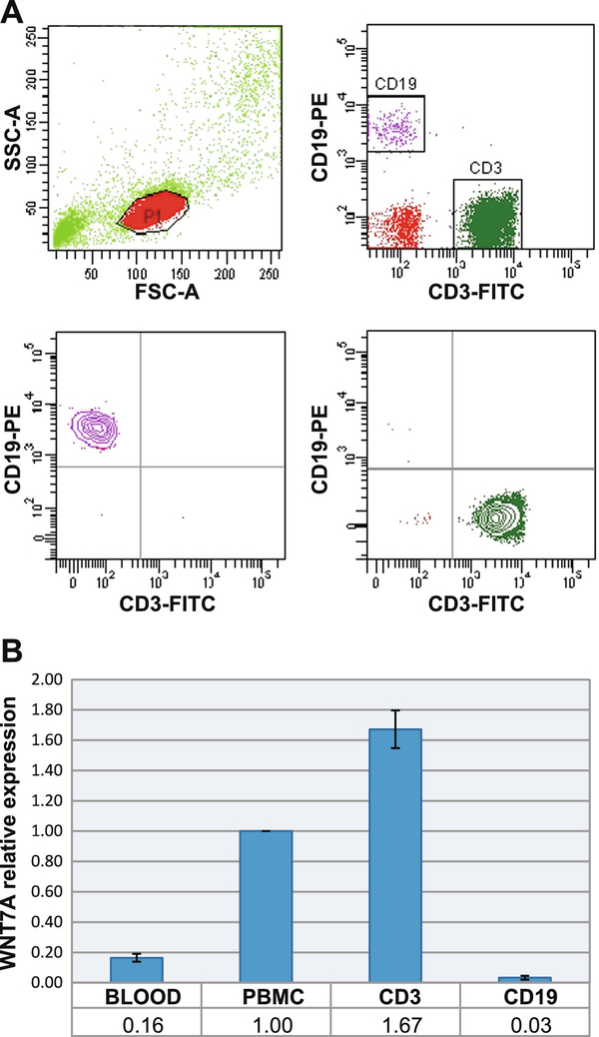
**Expression of *WNT7A *in total peripheral blood cells, Peripheral blood mononuclear cells (PBMC), sorted CD3^+ ^and CD19^+ ^cells obtained from healthy volunteers' samples**. (A) Dot blot graphics showing the selected region for sorting (upper left panel), CD3-Fluorescein isothiocyanate (FITC), and CD19-Phycoerythrin (PE) marked cells (upper right panel), isolated CD19^+ ^cells (lower left panel), and isolated CD3^+ ^cells (lower right panel). (B) Figure showing the average of the *WNT7A *relative expression ratio ± Standard deviation (SD) obtained from the different groups. Normalization was performed by setting CP values of PBMC as 1 and taking *GAPDH*, *RPL32*, and *RPS18 *as reference genes. Experiments were carried out at least twice in all cases.

The expression of *WNT7A *in normal mature blood cells is still not well characterized. From this starting point, we wanted to know whether normal peripheral blood cells express *WNT7A *and, more specifically, if T- or B-lymphocytes are expressing this Wnt ligand at the same level. In order to address this goal, we obtained cDNA from total blood cells, PBMC, T-lymphocytes (CD3^+ ^cells), and B-lymphocytes (CD19^+ ^cells)-positive cells. CD3^+ ^and CD19^+ ^cells were sorted by Flow cytometry (FCM) from healthy volunteers' PBMC (Figure 1A). Afterward, we determined the expression of *WNT7A *by qRT-PCR in the groups mentioned previously utilizing *GAPDH*, *RPL32*, and *RPS18 *as reference genes for relative expression analysis. Because efficiency and error of reactions are important for relative quantification analysis, standard curves obtained by serial dilutions (1:1, 1:2, 1:4, and 1:8) of cDNA were included in each PCR (see Additional File 1). Only data from PCR reactions showing an error < 0.04 and an efficiency of at least 1.8 were included in the analysis. Because CD3- and CD19-sorted cells were isolated from PBMCs, we normalized all values to those obtained in the PBMC. We found expression of *WNT7A *in total peripheral blood cells, but interestingly, we observed approximately 84% lower *WNT7A *expression in these cells than in PBMC (in which *WNT7A *relative expression was set as 1) (Figure 1B). Taking into account that 80% of peripheral blood cells are from myeloid origin and that 80% of PBMC are lymphoid, we assume that myeloid cells are expressing very low levels of *WNT7A *and that the main producers could be lymphoid cells. To delve deeper into this question, we analyzed the relative expression of *WNT7A *in pure CD3^+ ^and CD19^+ ^cells. We could determine that the highest expression of *WNT7A *corresponds to CD3^+ ^cells; in contrast, CD19^+ ^cells express nearly undetectable levels of this ligand. The *WNT7A *relative-expression difference observed between PBMC and CD3^+ ^cells correlates with the expected population's percentage of T-lymphocytes (~50-75%), B-lymphocytes (~10-25%), and NK cells (~5-20%) in PBMC. Summarizing these data, *WNT7A *expression rises to the greatest degree from CD3^+ ^cells.

### *WNT7A *expression is downregulated upon T-lymphocytes activation

**Figure 2:**
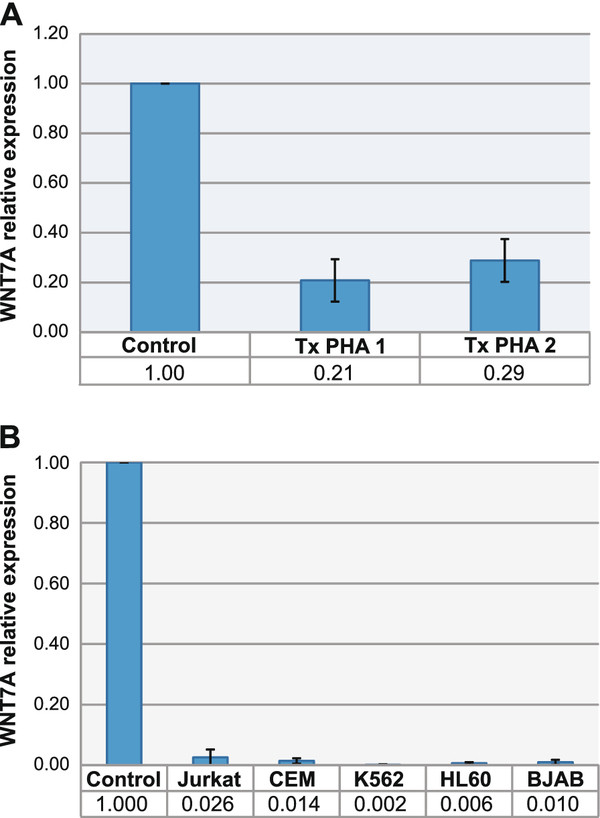
**Relative expression of *WNT7A *in control cells and leukemia-derived cell lines**. **(A) **Relative expression levels of *WNT7A *determined by qRT-PCR in resting and phytohemagglutinin-activated PBMC (activation was performed in PBMC from two volunteers and named TxPHA 1 and TxPHA 2). Values of the resting PBMCs (Control) of both volunteers were set as 1. **(B) **Relative expression levels of *WNT7A *determined by qRT-PCR in Jurkat, CEM, K562, HL60, and BJAB cell lines normalized to the healthy volunteers control group (setting as 1). In A, quantification was calculated using, Ribosomal Protein L32 (*RPL32*) and Ribosomal Protein S18 (*RPS18*) as reference genes. In B, quantification was calculated using additionally Glyceraldehyde-3-phosphate dehydrogenase (*GAPDH*). The graphics show means obtained with both reference genes and Standard deviations (SD). Experiments were carried out at least twice in duplicate.

Given that peripheral CD3^+ ^(mature resting lymphocytes) express *WNT7A*, we asked whether the expression of this gene is related with their resting non-proliferative state. To elucidate this question, PBMC were induced to proliferate by addition of Phytohemagglutinin (PHA - 2 μg/mL), a lectin molecule that preferentially induces T-lymphocytes proliferation. After 48 hours of PHA-treatment, total RNA was isolated, retro-transcribed and analyzed by qRT-PCR. As can be observed in Figure 2A, W*NT7A *mRNA levels were drastically affected upon PHA activation, decreasing from 1 (non-activated resting PBMC, control cells) to 0.21 and 0.29 (relative expression ratio). Amplification curves and melting peaks obtained for *WNT7A*, *RPL32*, and *RPS18 *in non-treated PBMC and PHA-treated PBMC are depicted in Additional File 2.

### Reduced *WNT7A *gene expression levels in leukemia-derived cell lines

Because *WNT7A *was expressed in resting peripheral T-lymphocytes, but severely reduced in activated T-lymphocytes, we assumed that leukemia-derived cell lines (which are undifferentiated and have a high grade of proliferation) should express low levels of this ligand. To test this hypothesis, we selected five different leukemia-derived cell lines: two of lymphoid origin (Jurkat and CEM); two of myeloid origin (K562 and HL60), and a lymphoma-derived B cell line (BJAB). We included also myeloid immature cell lines to determine whether this myeloid lineage indeed (as previously calculated by the analysis obtained from peripheral blood volunteer's samples) expresses very low levels of *WNT7A*. Therefore, we determined the quantitative expression of *WNT7A *by qRT-PCR assays. As a reference for comparison, we also included in this analysis a mix of cDNA obtained from total peripheral blood cells of five clinically healthy volunteers (control group). As can be observed in Figure 2B, relative expression utilizing *GAPDH*, *RPL32*, and *RPS18 *as reference genes exhibited very low expression levels of *WNT7A *in all leukemia-derived cell lines when compared with the control group. We observed relative values ranging from 0.026 in Jurkat cells to 0.002 in K562 cells when normalized to the control group (set as 1). After qRT-PCR assays, all amplified products were resolved in 1.5% agarose gels and visualized with Ultraviolet (UV) light for photo-documentation (data not shown).

In conclusion, the experiments performed previously allow us to demonstrate quantitatively that leukemia-derived cell lines of both myeloid and lymphoid origins express very low levels of *WNT7A*.

### Peripheral blood cells of patients with leukemia also showed a significant decrease in *WNT7A *gene expression

**Figure 3:**
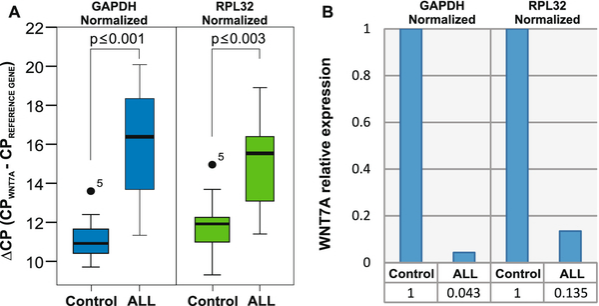
***WNT7A *expression in immature cells from patients with Acute lymphoblastic leukemia (ALL)**. **(A) **Box plot graphics showing ΔCP values taking *GAPDH *(left panel) or *RPL32 *(right panel) as reference genes. The graphics display median (dark lines), 25-75th percentile (boxes), interquartile ranges (whiskers), and outliers (small, dark circles) from 14 patients with ALL. Peripheral blood cells from 19 controls were included for comparison. Statistical significances are shown between both groups. **(B) **Graphic showing an average of the relative expression levels of *WNT7A *from the control and from the group with patients with ALL. The value obtained in the healthy volunteers control group was set as 1 and normalized either with *GAPDH or RPL32 *as reference genes.

After demonstrating that *WNT7A *expression is strongly reduced in leukemia-derived cell lines, we wanted to determine whether *WNT7A *expression could also be diminished in blood samples of patients with leukemia in comparison with clinically healthy volunteers. With this aim, the expression of *WNT7A *was analyzed in 14 samples of patients with Acute lymphoblastic leukemia (ALL) and in 19 samples of clinically healthy volunteers. We decided to determine the expression of *WNT7A *by normalizing to an internal reference gene; thus, the ΔCP was calculated, which represents a more absolute value. It is noteworthy that the ΔCP (CP of target gene - CP of reference gene) is inversely proportional to the expression of the target gene. Values of ΔCP of the analyzed groups (control and ALL) are represented in box plot graphics in Figure 3A. As can be observed in this figure, there is an evident difference between control values and those of patients with ALL normalized either with *GAPDH *(left panel) or with *RPL32 *(right panel). Higher ΔCP values were observed in the ALL group, which means lower expression of *WNT7A*. As shown in Figure 3A, when we compared the ALL group vs. the healthy volunteers group, we observed a statistical significance of *p *< 0.001 when normalized with *GAPDH *and *p *< 0.003 with *RPL32*. These results permit us to suggest that reduction of *WNT7A *expression could be a characteristic hallmark in patients with ALL.

Taking the median of the ΔCP values of controls and patients with ALL obtained with both reference genes, we calculated the average of *WNT7A *relative expression. As can be observed in Figure 3B, the average of *WNT7A *expression in patients in comparison to the control group (set as 1) diminished to 0.043 (*GAPDH*) or to 0.135 (*RPL32*).

In summary, there is a statistically significant decreased expression of *WNT7A*, not only in leukemia-derived cell lines in comparison with the control group, but also in patients with ALL when compared with healthy volunteers. Peripheral blood cells from four Acute myeloid leukemias (AML), three Chronic myeloid leukemia (CML), and five Chronic lymphocytic leukemia (CLL) also revealed a tendency to exhibit reduced *WNT7A *expression when compared with the control group (data not shown).

### Restoration of *WNT7A *inhibits cell growth in Jurkat cells

**Figure 4:**
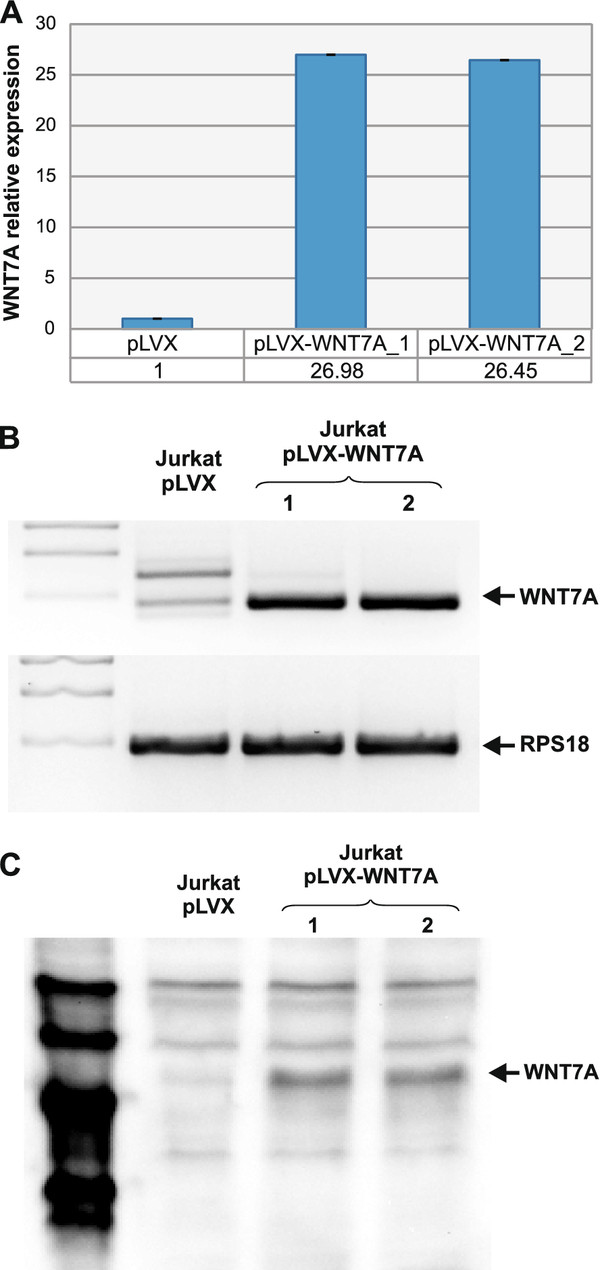
***WNT7A *overexpression in Jurkat cells**. Overexpression of *WNT7A *in Jurkat cells using the lentiviral system was confirmed by qRT-PCR assays. **(A) **Graphic showing *WNT7A *relative expression taking *GAPDH*, *RPL32 *and *RPS18 *as constitutive genes in cells infected with pLVX (vector alone) or with pLVX-WNT7A. Infection was carried out in duplicate (pLVX-WNT7A clone 1 and 2). **(B) **Amplification products of *WNT7A *and *RPS18 *were run in 2.0% agarose gels and photodocumented. **(C) **Western blot assays (from 50 μg total protein) showing the presence of WNT7a protein only in pLVX-WNT7A-infected cells. The magic mark (2 μl) was used as protein marker.

Because we found that *WNT7A *is expressed in CD3^+ ^resting lymphocytes derived from healthy volunteers and not in immature leukemia-derived cells, it was in our interest to discern the biological effect of WNT7a restoration on leukemia cells. For this purpose, we used lymphoblastic Jurkat cells as model and overexpressed *WNT7A *employing a lentiviral system (pLVX-Puro or pLVX-WNT7A-Puro; a detailed description is provided in Materials and Methods). After 48 h of infection, cells were cultured with 1 μg/mL puromycin for selection of infected cells, and RNA and protein extraction were conducted on day 7 to corroborate *WNT7A *overexpression by qRT-PCR and Western blot assays (Figure 4). After this selection time, *WNT7A *relative expression was measured in the pLVX empty-vector infected cells and in the pLVX-WNT7A clone 1- and -2-infected cells. We named these cells clone 1 and clone 2 because we infected Jurkat cells with the viral particles containing pLVX-WNT7A-Puro in two independent experiments. As can be observed in Figure 4A, expression of *WNT7A *in pLVX-WNT7A infected cells was increased nearly 27-fold when compared to the pLVX-empty vector. The amplified products from the qRT-PCR assays were also electrophoresed on 1.5% agarose gel as depicted in Figure 4B. Additionally, as can be observed in Figure 4C, W*NT7A *overexpression was also confirmed at protein levels.

**Figure 5:**
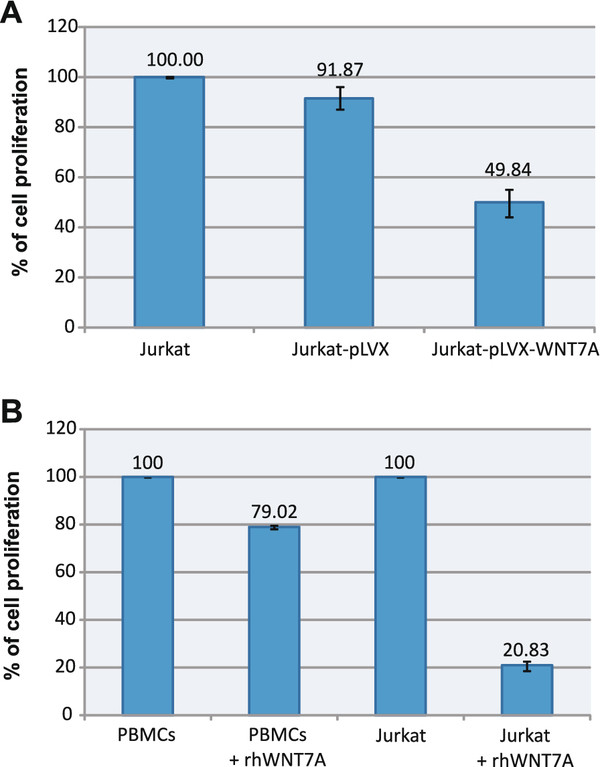
***WNT7A *inhibits cell proliferation in Jurkat cells**. **(A) **Percentage of cell proliferation measured in Jurkat cells, Jurkat-pLVX (stable transduced with empty-vector), or Jurkat-pLVX-WNT7A cells after 48 h of culture. Percentages of cell proliferation were obtained by taking Optical density (OD) values of the Jurkat cells as 100% cell proliferation. **(B) **Leukemia-derived Jurkat cells and PBMC were exposed for 48 h to 3 μg/mL of recombinant human WNT7a (+rhWNT7a). Afterward, the percentage of cell proliferation was measured by employing WST-1 and setting the value of non-treated control cells (Jurkat and PBMC for their respective assays) at 440 nm as 100% of cell proliferation.

To determine the biological effect of *WNT7A *restoration in cell proliferation, after 2 weeks puromycin selection, we seeded in the same number (10,000) of Jurkat, Jurkat-pLVX, and Jurkat-pLVX-*WNT7A *cells in 96-well plates and measured the percentage of cell proliferation after a 48 h culture. Percentages of cell proliferation were obtained by taking the Optical density (OD) values of Jurkat cells as 100%. As illustrated in Figure 5A, we observed a decrease in the rate of cell proliferation of Jurkat-pLVX cells (91.87%), but a significantly lower decrease was observed in Jurkat-pLVX-WNT7A cells (49.84%). This observation allowed us to speculate that the *WNT7A *produced by Jurkat-infected cells could somehow inhibit cell proliferation. To discern this point, Jurkat cells and resting PBMC obtained from one of the healthy volunteers were incubated with 3 μg/mL of commercially recombinant human WNT7a for 48 h. As can be observed in Figure 5B, incubation of Jurkat cells with recombinant human WNT7a (rhWNT7a) reduced their growth drastically after 48 h (to 20.83%). In contrast, the PBMC exhibited only a small effect, even reaching 79.02% of growth at 48 h.

### Restoration of *WNT7A *does not induce the canonical β-catenin pathway in Jurkat cells

**Figure 6:**
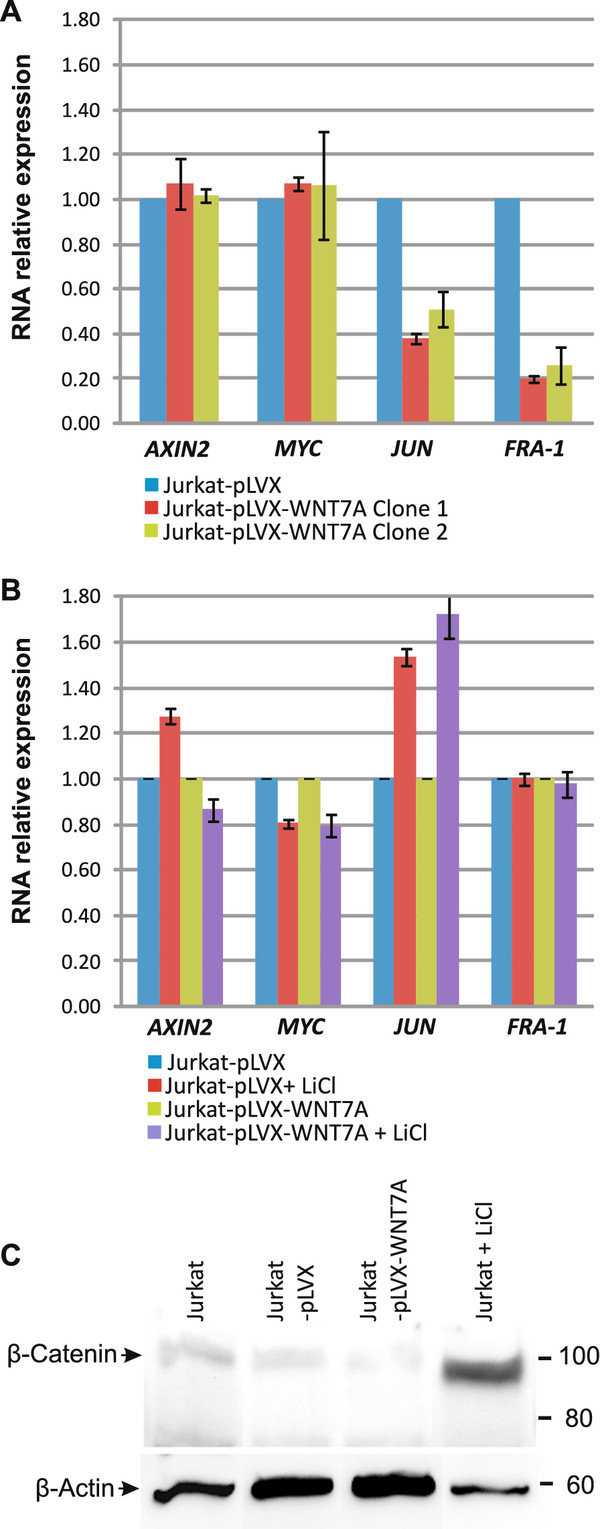
**Restoration of *WNT7A *does not induce the canonical β-catenin pathway in Jurkat cells**. **(A) **Relative expression levels of *AXIN2*, *MYC*, *JUN*, and *FRA-1*, determined by qRT-PCR in Jurkat-pLVX or Jurkat-pLVX-WNT7A clone-1 and -2 infected cells. Quantification was calculated by normalizing with Jurkat-pLVX (setting as 1) and utilizing *GAPDH *and *RPL32 *as reference genes. The graphic shows medians obtained with both reference genes ± Standard deviations (SD). **(B) **Relative expression levels of *AXIN2*, *MYC*, *JUN*, and *FRA-1*, determined by qRT-PCR in Jurkat-pLVX or Jurkat-pLVX-WNT7A clone-1 in the absence or presence of LiCl. Quantification was calculated by normalizing LiCl-treated cells with their corresponding Jurkat-pLVX or Jurkat-pLVX-WNT7A non-treated cells (setting each as 1) and utilizing *GAPDH *and *RPL32 *as reference genes. The graphic shows medians obtained with both reference genes ± SD. **(C) **Western blot analysis detecting β-catenin and β-actin in the presence or absence of LiCl and WNT7a.

To determine whether the proliferation inhibition in Jurkat cells observed after WNT7a restoration was mediated by activation of the canonical β-catenin pathway, we analyzed the expression of putative target genes for this pathway by qRT-PCR assays. For this purpose, we determined the mRNA expression of *AXIN2*, *MYC*, *JUN*, and *FRA-1 *in Jurkat-pLVX- and Jurkat-pLVX-WNT7A-infected cells clone 1 and 2. As can be appreciated in Figure 6A, none of the aforementioned genes was activated by *WNT7A *restoration. Instead, decreased expression in *JUN *and *FRA-1 *was observed in clones expressing *WNT7A*. Interestingly, when we incubated cells with LiCl (0.01 M) for 20 h (it is known that LiCl activates Wnt signaling by inhibiting GSK3β, leading to β-catenin stabilization and translocation into the nucleus [25,26]), a restoration of the expression levels of *JUN *and *FRA-1 *was observed, which indicates that LiCl antagonize WNT7A activity in these cells (see Figure 6B). Additionally, to support that WNT7a in this model did not induce the canonical pathway, we performed Western blot assays to detect β-catenin in Jurkat, Jurkat-pLVX, and Jurkat-pLVX-WNT7A cells. As control, we also included LiCl-treated Jurkat cells. As expected, accumulation of β-catenin was only observed in the LiCl-treated cells and not in the *WNT7A *expressing cells (Figure 6C). These results clearly support the idea that the canonical pathway is not involved in the action of *WNT7A *on Jurkat cells.

### Restoration of *WNT7A *inhibits cell growth in K562, BJAB, and CEM cells

**Figure 7:**
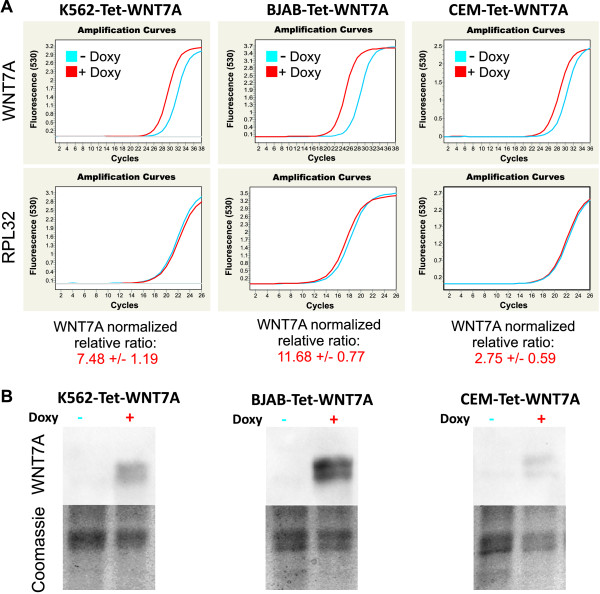
***WNT7A *overexpression in K562-, BJAB- and CEM-Tet-WNT7A cells**. Overexpression of *WNT7A *in these cell lines using the doxycycline-inducible lentiviral expression system was confirmed by qRT-PCR and Western blot assays. **(A) **Graphics showing the amplification curves obtained for *WNT7A *and *RPL32 *in cells treated or not with Doxycycline (Doxy) for 4 h. The *WNT7A *normalized relative ratio of the cells treated with Doxy was calculated utilizing the non-treated cells as calibrator and taking *GAPDH, RPL32 *and *RPS18 *as reference genes in two independent experiments. **(B) **Western blot assays (from 50 μg total protein) showing the presence of WNT7a protein after a 38-h incubation with Doxy. Photo-documentation of the Coomassie stained gel is included as protein loading control.

To confirm that restoration of *WNT7A *inhibits cell growth in leukemia-derived cells, we modified K562, BJAB, and CEM cells to overexpress *WNT7A *employing an inducible-lentiviral expression system (as described in Methods). We first obtained K562-, BJAB-, and CEM-Tet cells that contained only the regulator vector (Tet). Next, we obtained K562-, BJAB-, and CEM-Tet-WNT7A cells that should express *WNT7A *in the presence of doxycycline. To verify *WNT7A *overexpression, K562-, BJAB-, and CEM-Tet-WNT7A cells were grown in the absence or presence of doxycycline (750 ng/mL) for 4 h. Afterward, RNA extraction and retrotranscription were performed for the qRT-PCR assays. As can be observed in Figure 7A, expression of *WNT7A *indeed increased in all cell lines, rising 7.48 ± 1.19-fold in K562, 11.68 ± 0.77-fold in BJAB and 2.75 ± 0.59-fold in CEM. This induced overexpression was also confirmed at protein levels (after 48 h of doxycycline exposure) by Western blot analysis (see in Figure 7B).

**Figure 8:**
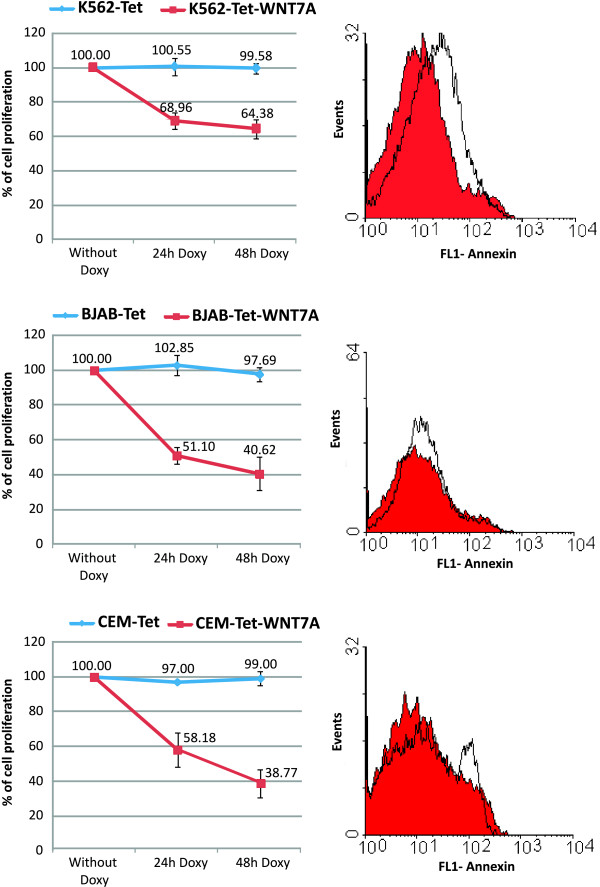
**Restoration of *WNT7A *in K562-, BJAB-, and CEM-Tet-WNT7A cells inhibits their cell proliferation**. Graphs of the left column depict the percentage of cell proliferation measured in K562-, BJAB-, and CEM-Tet-WNT7A cells after 24-h and 48-h incubation with Doxycycline (Doxy). The empty-vector cell lines (K562-, BJAB-, and CEM-Tet) were also included as controls. The percentage of cell proliferation was measured by adding WST-1 to the cell cultures for 2 h and by reading the absorbance of treated and untreated cells at 440 nm. The value in each cell line of non-treated cells after 24 or 48 h was setting as 100% of cell proliferation. Right column graphs illustrate the histograms of the flow-cytometric analysis done for apoptosis detection utilizing Annexin-V-FLUOS as markers in non-treated (filled curve) and 48-h Doxy-treated cells (open curve). Ordinate: number of cells; Abscissa: fluorescence intensities.

Once we could establish that our inducible-WNT7A system was working adequately, we determined the *WNT7A *restoration effect on cell proliferation. For this purpose, we added WST-1 to the culture cells growing in the presence or absence of doxycycline after 24 and 48 h of incubation. Percentages of cell proliferation were obtained by taking the OD values of the cells growing in the absence of doxycycline as 100% of cell proliferation. As may be seen in Figure 8, WNT7A*-*inducible expression in all three cell lines decreased strongly, especially in BJAB-Tet-*WNT7A *and in CEM-Tet-WNT7A cells, decreasing to 40.62 and 38.77%, respectively, after 48 h of growth in the presence of doxycycline.

## Discussion

Wnt signaling is conserved from invertebrates to vertebrates and regulates early embryonic development, as well as the homeostasis of adult tissues; as a central pathway in both physiological processes, dysregulation of Wnt signaling is associated with many human diseases, and particularly with cancer [1]. Recently, Wnt signaling has also been implicated in hematopoiesis, both in self-renewal and in differentiation [1,10,18]. Based on these observations, it is hypothesized that dysregulation of the WNT pathway might contribute to the pathogenesis of lymphoproliferative diseases [12].

Despite the modest number of reports on the potential roles of Wnt signaling in leukemia, it is increasingly clear that Wnt signaling is dysregulated in several types of leukemia [12,18,27]. Some of these findings involve over- or underexpression of several Wnt ligands or Frizzled receptors [16,19,28,29], hypermethylation of natural WNT inhibitors [30], and overexpression of β-catenin [21].

Despite this knowledge, there are a very limited number of publications on the expression of *WNT7A *and its role in the biology of blood cells. One of the first observations of the implication of *WNT7A *in hematological disorders was the frequent deletion of the 3p25 chromosome band observed in patients with AML, CML, and ALL [31]. As is known, *WNT7A *is also localized at this chromosomal region [32,33] and its deletion could be an important step during the neoplastic transformation.

In this paper, we report the expression of *WNT7A *in normal peripheral T-lymphocytes and strongly reduced *WNT7A *expression, not only in leukemia-derived cell lines, but also in the peripheral blood cells of patients with leukemia.

We were able to demonstrate that T-lymphocytes, but not B-lymphocytes, express *WNT7A *(ΔCP 11.47 ± 1.2). In agreement with this observation, Lu et al. also found expression of this ligand in peripheral blood lymphocytes (ΔCP 11.81 ± 0.99), but do not determined that this expression was afforded mainly from T-cells [19]. In contrast, Sercan et al. found *WNT7A *expression in both T- and B-cells obtained from healthy volunteers [23]. Discrepancies in these data could be due to the different method employed for quantification. The previously mentioned research group quantified *WNT7A *expression by comparing the densities of amplified *WNT7A *and β-actin PCR products visualized on agarose gels, while our group did this by performing qRT-PCR assays, which afford very precise data for quantification analysis.

We found from 38- to 500-fold lower expression in leukemia-derived cell lines than in healthy control cells (see Figure 2B). These results are in agreement as reported recently by Sercan et al., in which they did not find *WNT7A *expression in leukemia-derived cell lines K562, HL60, Jurkat, and Namalwa [23]. However, the authors measured qualitatively, while we determined *WNT7A *expression quantitatively.

On the other hand, expression of *WNT7A *in hematological diseases has been only determined in patients with CLL and AML. Memarian et al. observed reduced expression of *WNT7A *in Iranian patients with AML compared with normal subjects [24]; however, the authors did not find this difference in patients with CLL [29]. It is noteworthy that in both of these previously mentioned reports, *WNT7A *expression was calculated using the band densities of *WNT7A *and β-actin after conventional PCR. In agreement with the results of Memarian et al. we also observed reduced expression of *WNT7A *also in patients with AML, but statistical significance was not reached, probably due to the low number of patients with AML whom we analyzed (data not shown). Regarding expression of this ligand in patients with CLL; Lu et al. also observed lower *WNT7A *expression in patients with CLL (ΔCP 15.43 ± 2.94) when compared with healthy peripheral blood lymphocytes (11.81 ± 0.99)[19]. In this sense, we also observed this behavior in 4 out of 5 CLL patients (ΔCP 16.3 ± 1.5). Despite this low number of CLL patients, we found a statistical significance of *p ≤0.02 *when compared with healthy control cells (ΔCP 11.47 ± 1.2) (data not shown).

Interestingly, when we analyzed peripheral blood cells from 14 patients with ALL, these also expressed reduced *WNT7A *expression (ΔCP 15.19 ± 2.5) and we found a statistically significant difference of *p ≤0.001 *(*GAPDH*) and *p ≤0.003 *(*RPL32*) when compared with the control group (Figure 3).

Another important observation that we discerned is that *WNT7A *decreases acutely after PHA activation. To our knowledge, this is the first report evidencing that lymphocytes require reduction of their *WNT7A *levels in order to proliferate and suggests that dysregulation in the expression of this ligand needs to occur during oncogenesis to lose control of cell proliferation. Interestingly, it has been reported that T-cell activation by phytohemagglutinin results in a strong increase of phosphorylated GSK3β [34], which in turn targets beta-catenin for ubiquitylation and proteasomal degradation [35].

With respect to the reference genes used in the qRT-PCR assays, it is important to mention that there are no perfect reference genes for every treatment in every cell line. Thus, we used at least two reference genes in each assay and also evaluated some samples with a total of five reference genes (please see Additional Files 1 and 2). It has been determined that one of the reference genes that we used (RPS18) is useful as internal control for quantitative PCR in human lymphoblastoid cells, because constant levels of expression across the cell lines used were found following exposure to ionizing radiation as well as to PHA [36]. However, it could be that some, but not all, of the changes in *WNT7A *expression may be caused by changes in reference-gene expression when cells were treated with PHA.

To our knowledge, no other papers relating *WNT7A *and leukemia have been published; however, reduced or absent expression of *WNT7A *has also been observed in lung cancer when compared with normal lung and mortal, short-term bronchial epithelial culture by qRT-PCR assay [36,37].

Furthermore, it has been reported that WNT7a activates E-cadherin expression in lung cancer cells and that *WNT7A *loss may be important in lung cancer development or in progression due to its effects on E-cadherin, because E-cadherin in cancer has been associated with dedifferentiation, invasion, and metastasis [38]. In addition to the role of *WNT7A *observed in leukemia and lung cancer, disruptions or alterations of the *WNT7A *gene have also been found in oral premalignant lesions [39] and in esophageal squamous cells [40].

We were also able to demonstrate, in the Jurkat leukemia-derived cell line, that restoration of *WNT7A *(by lentiviral overexpression or the addition of human recombinant protein) inhibits cell proliferation (Figure 5). Moreover, with the inducible-lentiviral overexpression system, we also confirmed this observation in K562, BJAB, and CEM cells after WNT7a expression; however, induction of cell death was not observed (Figure 8). Spinsanti et al. also reported an anti-proliferative action of WNT7a expression in undifferentiated PC12 cells [41]. Additionally, recent studies have demonstrated that the combined expression of *WNT7A *and *Frizzled 9 *(Fzd9) in Non-small cell lung cancer (NSCLC) cell lines inhibits transformed growth by activating ERK5 and increasing PPARgamma activity, representing a novel tumor suppressor pathway in lung cancer [36,42,43]. However, the biological role of *WNT7A *action in cancer is controversial at present; some evidence supports its activity as an oncogene, but there is also evidence of its tumor suppressor action [36,44,45]. This dual role can be explained by the FZD proteins that bind WNT7a. It has been reported that the binding of WTN7a and FZD5 induces the canonical pathway, which has been related with cancer development [46,47]. On the other hand, WTN7a can also bind FZD-10 and -9, which in turn activated the c-Jun NH2-terminal kinase pathway (JNK). Activation of JNK has been shown to antagonize the canonical pathway [47].

Due to the reported dual behavior of *WNT7A *as a cell-proliferation inducer or blocker, it is reasonable to think that Jurkat cells preferentially express anti-proliferative FZD partners of *WNT7A*. To address this question, we analyzed the presence of FZD mRNAs in Jurkat cells compared with T-lymphocytes from healthy controls and found overexpression of FZD-3 and -6 and downmodulation of FZD-5 and -10 in Jurkat cells (data not shown). On analyzing hematopoietic cells and leukemia-derived cells, Sercan et al. also found expression of FZD-3 and -6 in leukemia-derived T-lymphocytes [23]. The presence of FZD-6 in lymphocytes is interesting, because it has been shown that FZD-6 can act as a negative regulator of the canonical pathway [48]. Whether FZD-6 can interact with WTN7a in lymphocytes and what the biological consequences of this interaction would be are questions that remain open.

It has been observed that increased expression of some WNT ligands such as WNT3a, induces activation of the canonical pathway, accompanied by an increase in the proliferation and survival of leukemia cells [49]. In addition, it has been reported that β-catenin comprises an integral part of AML cell proliferation and cell cycle progression [50]. Because we observed downmodulation of *WNT7A *in leukemia-derived cells, it appears that WNT7a in Jurkat cells does not activate the WNT/β-catenin pathway. Evidence that supports this notion is the finding that mRNA levels of the putative canonical target genes *AXIN2, MYC, JUN*, and *FRA-1 *were not increased after WNT7a restoration (see Figure 6). In contrast, mRNA from *JUN *and *FRA-1 *were strongly downregulated in *WNT7A-*expressing cells, but again restored (*FRA-1*) or even upregulated (*JUN*) when cells were treated with LiCl (Figures 6A &6B). Concerning this point, it has been reported that the β-catenin - T cell-factor/lymphoid-enhancer-factor complex directly interacts with the promoter region of *JUN *and *FRA-1 *[51]. Because we observed restoration of the expression levels of *JUN *and *FRA-1 *after LiCl treatment, it is very probable that LiCl antagonize WNT7a activity in these cells. An additional observation that supports the idea that WNT7a is working in this model in a non-canonical pathway is that expression of β-catenin was not increased after WNT7a restoration (as seen in Figure 6C).

## Conclusions

In conclusion, we are demonstrating, by qRT-PCR analysis, that *WNT7A *is significantly reduced in leukemia-derived cell lines as well as in patients with leukemia when compared with clinically healthy volunteers. The finding that *WNT7A *restoration inhibits proliferation of leukemia-derived Jurkat cells, but not of PBMC, allows us to assume that *WNT7A *can be acting as a modulator of cell proliferation, especially in T-cells that are producing this protein. In this regard, impairment of this anti-proliferative function could be an important event in leukemia and in some cancer-cell types. If this is true, therapeutic tools directed toward restoring *WNT7A *expression in patients with leukemia might increase the probabilities of their overcoming this disease.

## Competing interests

The authors declare that they have no competing interests.

## Authors' contributions

AAL wrote the paper and supervised all experiments performed as principal investigator; JARA and EBC recruited patients, collected samples, and isolated RNA; ABOH performed all qRT-PCR assays, Western blots, and PHA treatments, and aided in writing the manuscript; IDMC cloned, sequenced, and performed *WNT7A *overexpression with the Lentiviral system; BGC carried out the sorting assays, collaborated in the detection of WNT7a and contributed in the PHA treatments; MRS performed *WNT7a *overexpression with the inducible-Lentiviral system, conducted the assays with the recombinant protein, and performed growth inhibition analysis; MARR and PBN aided in writing and designing the protocol and also analyzed data; PCOL and GHF detected the apoptosis rate by FACS; AAL, LFJS, and ABC performed the statistical analysis, and AAL and LFJS conceived of and discussed the experimental work. All participants contributed commentary on and corrected the manuscript.

## Pre-publication history

The pre-publication history for this paper can be accessed here:

http://www.biomedcentral.com/1471-2407/12/60/prepub

## Supplementary Material

Additional file 1 **Relative expression analysis comparison among five of the most used reference genes**. A panel of five different reference genes was used to calculate *WNT7A *expression. Amplification curves of all genes are shown in BJAB cells treated or not with Doxycycline (+Doxy). Standard curves were performed to calculate error and efficiency in all genes used. Table show *WNT7A *relative expression normalized with the different reference genes. Click here for file

Additional file 2 **Amplification curves obtained in cells treated or not with PHA**. Upper panel graphics shown the Amplification curves obtained for *WNT7A *in non-treated PBMC and in PBMC treated for 48 h with PHA. Middle graphics correspond to *RPS18 *and bottom panel graphics to *RPL32*. Melting peaks are also shown in the right panels, which indicate the specificity of the PCR reaction. Click here for file
