# Supplementary material for: Peripheral T-lymphocytes express WNT7A and its restoration in leukemia-derived lymphoblasts inhibits cell proliferation
Source: BMC Cancer. 2012 Feb 7;12:60. doi: 10.1186/1471-2407-12-60 (PMC3299642; doi:10.1186/1471-2407-12-60)
Supplement: Additional file 1 — Relative expression analysis comparison among five of the most used reference genes. A panel of five different reference genes was used to calculate WNT7A expression. Amplification curves of all genes are shown in BJAB cells treated or not with Doxycycline (+Doxy). Standard curves were performed to calculate error and efficiency in all genes used. Table show WNT7A relative expression normalized with the different reference genes. [file 1471-2407-12-60-S1.PDF]

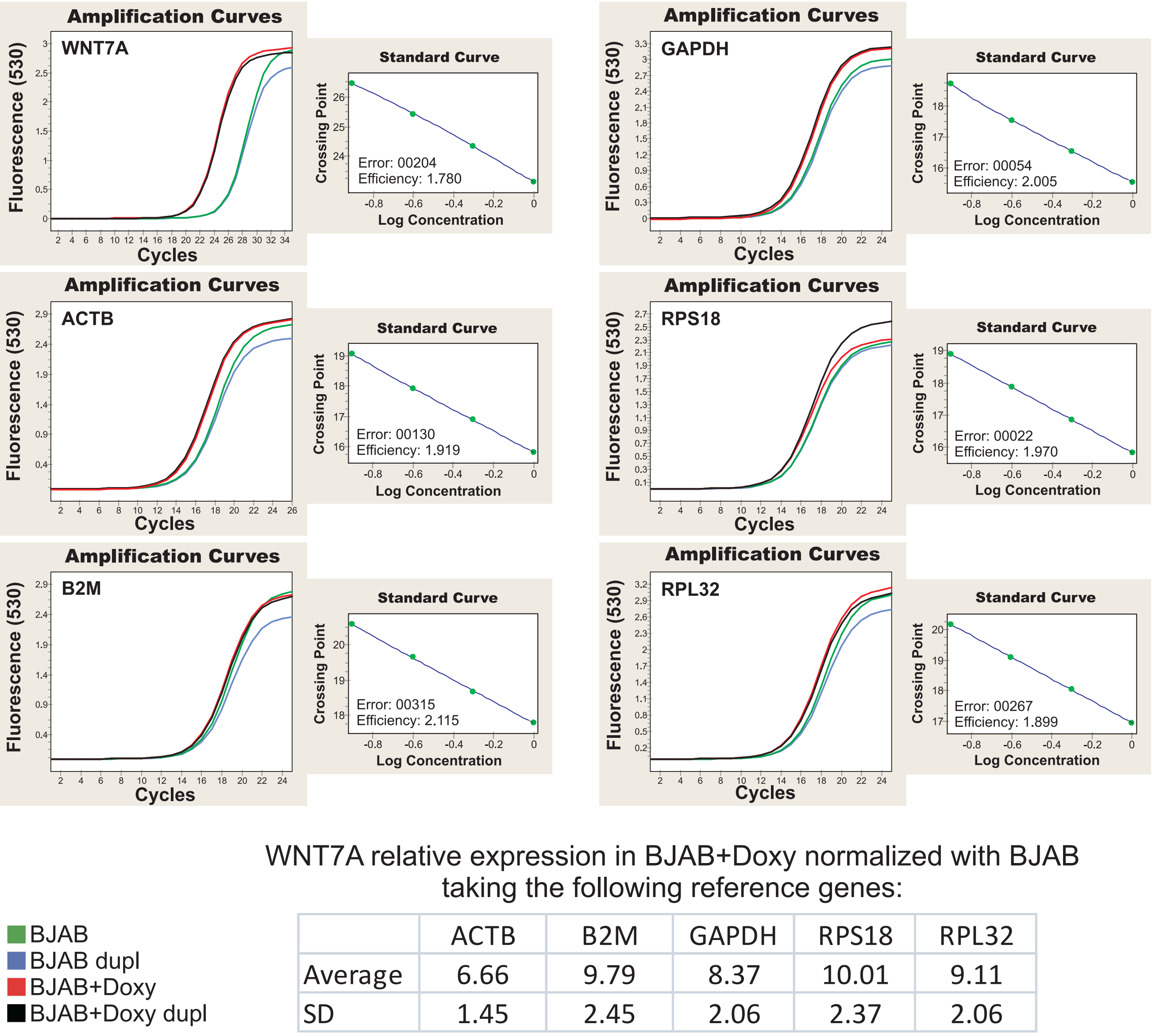

WNT7A relative expression in BJAB+Doxy normalized with BJAB taking the following reference genes:

	ACTB	B2M	GAPDH	RPS18	RPL32
Average	6.66	9.79	8.37	10.01	9.11
SD	1.45	2.45	2.06	2.37	2.06
