# Supplementary material for: Peripheral T-lymphocytes express WNT7A and its restoration in leukemia-derived lymphoblasts inhibits cell proliferation
Source: BMC Cancer. 2012 Feb 7;12:60. doi: 10.1186/1471-2407-12-60 (PMC3299642; doi:10.1186/1471-2407-12-60)
Supplement: Additional file 2 — Amplification curves obtained in cells treated or not with PHA. Upper panel graphics shown the Amplification curves obtained for WNT7A in non-treated PBMC and in PBMC treated for 48 h with PHA. Middle graphics correspond to RPS18 and bottom panel graphics to RPL32. Melting peaks are also shown in the right panels, which indicate the specificity of the PCR reaction. [file 1471-2407-12-60-S2.PDF]

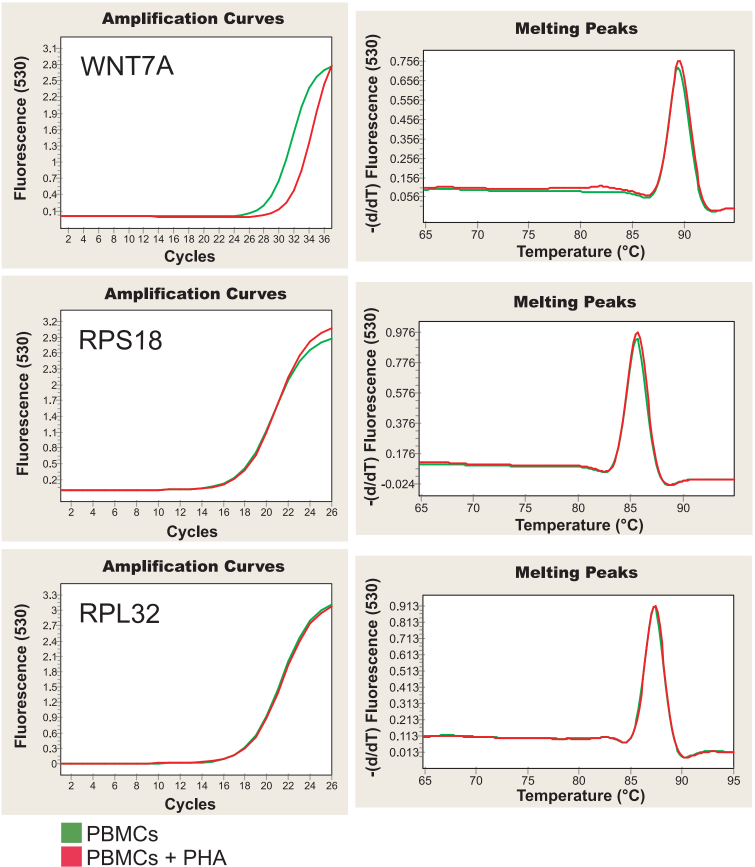
Amplification Curves
WNT7A
Fluorescence (530)
3.1
2.8
2.5
2.2
1.9
1.6
1.3
1
0.7
0.4
0.1
2 4 6 8 10 12 14 16 18 20 22 24 26 28 30 32 34 36
Cycles
Melting Peaks
-(d/dT) Fluorescence (530)
0.756
0.656
0.556
0.456
0.356
0.256
0.156
0.056
65 70 75 80 85 90
Temperature (°C)
Amplification Curves
RPS18
Fluorescence (530)
3.2
2.9
2.6
2.3
2
1.7
1.4
1.1
0.8
0.5
0.2
2 4 6 8 10 12 14 16 18 20 22 24 26
Cycles
Melting Peaks
-(d/dT) Fluorescence (530)
0.976
0.776
0.576
0.376
0.176
-0.024
65 70 75 80 85 90
Temperature (°C)
Amplification Curves
RPL32
Fluorescence (530)
3.3
3
2.7
2.4
2.1
1.8
1.5
1.2
0.9
0.6
0.3
0
2 4 6 8 10 12 14 16 18 20 22 24 26
Cycles
Melting Peaks
-(d/dT) Fluorescence (530)
0.913
0.813
0.713
0.613
0.513
0.413
0.313
0.213
0.113
0.013
65 70 75 80 85 90 95
Temperature (°C)
PBMCs
PBMCs + PHA
